# Integrated live imaging and molecular profiling of embryoid bodies reveals a synchronized progression of early differentiation

**DOI:** 10.1038/srep31623

**Published:** 2016-08-17

**Authors:** Jonathan Boxman, Naor Sagy, Sirisha Achanta, Rajanikanth Vadigepalli, Iftach Nachman

**Affiliations:** 1 Department of Biochemistry and Molecular Biology, Tel Aviv University, Israel; 2 Department of Pathology, Anatomy and Cell Biology, Thomas Jefferson University, Philadelphia, PA, USA

## Abstract

Embryonic stem cells can spontaneously differentiate into cell types of all germ layers within embryoid bodies (EBs) in a highly variable manner. Whether there exists an intrinsic differentiation program common to all EBs is unknown. Here, we present a novel combination of high-throughput live two-photon imaging and gene expression profiling to study early differentiation dynamics spontaneously occurring within developing EBs. Onset timing of Brachyury-GFP was highly variable across EBs, while the spatial patterns as well as the dynamics of mesendodermal progression following onset were remarkably similar. We therefore defined a ‘developmental clock’ using the Brachyury-GFP signal onset timing. Mapping snapshot gene expression measurements to this clock revealed their temporal trends, indicating that loss of pluripotency, formation of primitive streak and mesodermal lineage progression are synchronized in EBs. Exogenous activation of Wnt or BMP signaling accelerated the intrinsic clock. CHIR down-regulated Wnt3, allowing insights into dependency mechanisms between canonical Wnt signaling and multiple genes. Our findings reveal a developmental clock characteristic of an early differentiation program common to all EBs, further establishing them as an *in vitro* developmental model.

The coordinated progression of different cell lineages is essential for the formation of functional tissues and organs. Recent efforts have been focused on developing and optimizing *in vitro* platforms to study the mechanisms underlying stem cell differentiation as well as multi-lineage progression, using two- and three-dimensional cultures^1^,^2^,^3^,^4^. Better definition of the constraints on multi-lineage differentiation processes in these systems would enhance their use in the study of development and developmental defects.

Embryonic stem cells can be aggregated into embryoid bodies (EBs), which have the potential to differentiate to a diverse population of adult specialized cells^2^,^5^,^6^,^7^,^8^. Even though EBs differentiate in a less strictly defined fashion than embryos, they display embryogenesis-like processes such as germ layer formation, ECM secretion, and primitive streak formation^2^,^6^,^7^,^8^. The timing and pattern of these processes are influenced by a profusion of variables such as medium composition, growth surfaces and physical constraints^2^. For example, initial EB size affects the extent of mesodermal (and subsequently cardiac) vs. ectodermal differentiation^9^. The differentiation of EBs into cells of the three germ layers, even in the absence of externally added directive signals, indicates that the required signals for these processes can autonomously build up within each EB. BMP, Wnt, FGF and activin signaling pathways have all been shown to play important roles in inducing these transitions, both *in vivo* and *in vitro*^10^,^11^,^12^. FGF signaling was shown to be essential for the onset of neural ectoderm markers^13^,^14^. Wnt and BMP signaling components are essential for the establishment of primitive streak and mesendoderm formation and, in EBs, influence mesendoderm differentiation and axis formation^15^,^16^,^17^. How these signals interact to coordinate the growth and relative composition of multiple lineages is not fully characterized. EBs can serve as a valuable tool for studying cell to cell signaling mediated development, and are particularly compatible with high-throughput analysis of post-implantation differentiation processes.

During differentiation, EBs contain a temporally-shifting mixture of various cell lineages, each with its own gene expression profile, signaling characteristics, and lineage history. Individual EBs sampled at a particular time may therefore be comprised of multiple unsynchronized lineages at discrete differentiation stages, such that sampling of pooled EBs would span these multilayered spectra and mask critical transitions and relationships. Analysis based on snapshot images of developmental markers at specific time points lacks temporal perspective and cannot differentiate between alternative explanations of observed expression patterns, e.g. whether a marker remains unexpressed because it is lagging or because it was never induced, whether it is at its peak expression, or whether it is spatially expanding or contracting, possibly leading to erroneous conclusions. Alternatively, live imaging EBs with fluorescently labeled genes is limited to a few spectrally distinguishable fluorescent markers at a time. Expression profiling methods measure a snapshot of multiple genes, providing a fuller picture of the molecular states, but only for a single time point per EB due to their sample-destructive nature. Recently, several methods were proposed to define a “pseudotime” from single cell expression measurements based on the distribution of gene expression values sampled from a dynamic process^18^,^19^,^20^. While such methods can provide self-consistent expression trends, they do not in general reveal directionality nor real time estimates of the rate of changes taking place. Furthermore, they require large amounts of densely sampled data points. A temporally distinguishable marker longitudinally measured in each sample could provide this additional information, without requiring the vast amount of samples needed for robust inference of ”pseudotime”.

Here we combine live 3D imaging with molecular profiling of single EBs to study mesendoderm through early mesoderm differentiation in embryoid bodies. We use Brachyury (Bry-GFP) as a mesendodermal differentiation marker^21^,^22^,^23^ and show that EBs display remarkably uniform Bry-GFP dynamics. We characterize these dynamics and define a developmental clock for each EB. Using this clock we temporally map gene expression trends from Bry-GFP onset, characterizing a wild type differentiation progression model in EBs. We evaluate the effect of exogenous Bmp4 or canonical Wnt pathway activation on the dynamics of mesendoderm lineage specification, finding that both lead to earlier mesendodermal onset and expedited transitions to later differentiation stages. Finally, by exploiting an unexpected dual effect of CHIR, which both activated the canonical Wnt pathway and inhibited Wnt3 expression, we distinguish between different modes of Wnt regulation for multiple genes. Our integrated live imaging and molecular profiling approach establishes development-like mesendodermal dynamics in early stage embryoid bodies, supporting their use as models for early developmental processes.

## Results

### Mesendoderm induction progresses in a common pattern in EBs

**Figure 1:**
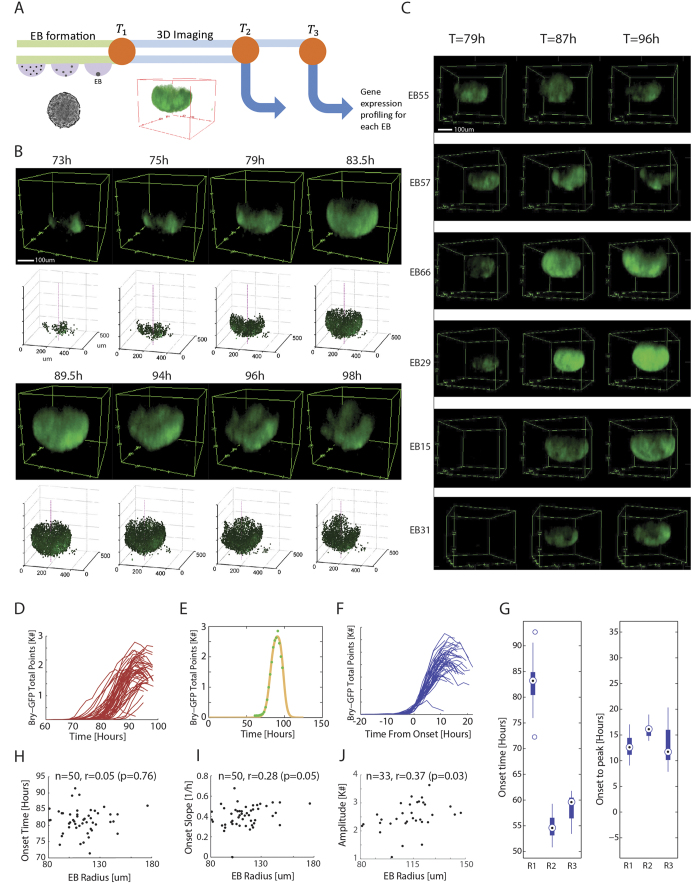
Brachyury-GFP progresses in a prototypical spatio-temporal pattern with high onset variability in EBs. (**A**) Experimental approach flow schematics. Brachyury-GFP EBs are live-imaged over 2–3 days, then individually collected for expression profiling at a selected time point. (**B**) Progression of Brachyury-GFP expression in an EB undergoing non-directed differentiation, with its corresponding segmentation data. (**C**) 3D visualization of six EBs at three different time points, demonstrating Bry-GFP onset variability. (**D**) Total number of Bry-GFP points as a function of time for 51 EBs imaged in one experiment. Bry-GFP onset shows a spread of >20 hours. (**E**) A simple impulse model describing Bry-GFP dynamics (yellow line) fit to data from a single EB (green). (**F**) The dynamic profiles of the EBs shown in (d) aligned by their onset time, defined by the impulse model. (**G**) Distributions of Bry-GFP onset and onset to peak time for three different experiments (onset: n = 50, 10, 12, onset to peak: n = 15, 10, 12 since not all EBs from repeat 1 reached peak expression). (**H**–**J**) Bry-GFP onset time (**H**), onset slope (**I**) or amplitude (**J**) plotted against EB radius prior to onset, showing no significant correlation in any of the three parameters.

To examine the dynamics and consistency of early differentiation within EBs, we analyzed the spatio-temporal pattern of mesodermal lineage induction using a Brachyury-GFP reporter. Mouse Brachyury-GFP E14 ES cells^24^ were suspended in differentiation medium, aggregated into EBs in hanging drops for 24/48 hours and then seeded in microwell arrays. We live-imaged 30–100 EBs in each experiment by two-photon microscopy over a period spanning the time prior to Brachyury-GFP onset to its eventual decline, and the output data was spatially segmented and analyzed (Fig. 1A). Microwell diameter (400 um) was calibrated so that by the end of the experiment most of the EBs were still not spatially confined, in order to avoid differentiation effects stemming from mechanical strain^9^,^25^. Through this optimized approach, we obtained unique high-throughput, high-resolution data sets on Brachyury dynamics in hundreds of EBs along 30–70 hours of differentiation, sampled every 90–120 minutes.

We found that EBs display a highly consistent spatial pattern of Brachyury expression. With almost no exceptions, Bry-GFP expression onset began from a single locus and spread from it over the EB’s outer shell. After a certain period the signal receded, typically toward the direction of the original locus, which remained Bry-GFP positive for the longest time period (Fig. 1B, Supplementary Movie 1, Supplementary Fig. 1). EBs displayed a large temporal variability in the time of Bry-GFP onset (Fig. 1C,D, Supplementary Movie 2). To obtain a more robust estimate of onset and peak times and to filter measurement noise, we fitted the total Bry-GFP curves to a simple kinetic model (Fig. 1E, see Methods). EBs that were seeded together became Bry-GFP positive between 72.3 and 92.7 hours after seeding (83.3 ± 3.7 hours), while the interval from onset to peak expression measured 12.8 ± 2.2 hours over the same EBs (Fig. 1F). When comparing different repeats of the experiment, the mean onset time varies by more than 20 hours, but the intra-experiment variability of the onset time, as well as the onset-to-peak interval, were similar across experiments (Fig. 1G). Inter EB variation might stem from varying environmental conditions, from stochastic events in an EB, or from both. However, once Bry-GFP is expressed, its spatio-temporal progression is constrained to common dynamics, independent of onset timing^26^. Such behavior is consistent with differentiation along a conserved trajectory but at different kinetic rates, rather than variation in the regulatory programs that would lead to distinct dynamics of Bry-GFP.

In contrast to single cell colonies, EBs are aggregated from a large number of cells (in our system 500 on average), with some variability in starting cell number. This variability, resulting in a span of initial EB sizes, could underlie the observed differences in mesodermal progression^9^. We measured the EB sizes prior to Bry-GFP onset, at 60 hours, and found that though the radius varied substantially (113 ± 19 um, span: 81–176 um), neither the onset of Bry-GFP nor its progression rate showed significant correlation to EB size before onset (Fig. 1H,I). Bry-GFP amplitude is weakly correlated to EB size for EBs smaller than approximately 100 um, as the GFP signal can fill the whole EB, while for larger EBs there is no such dependence (Fig. 1J). Together, these observations suggest the dynamics of Brachyury onset and progression are independent of the EB size.

### Early lineage genes correspond with a Bry-GFP clock

**Figure 2:**
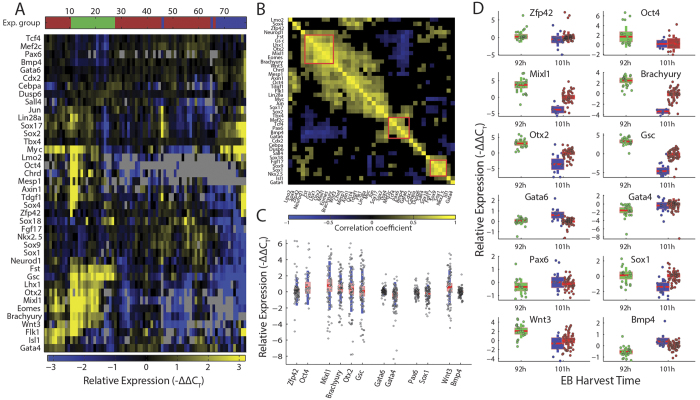
EB gene expression shows functional clusters and a wide spread. (**A**) Clustered expression of 40 genes in 78 EBs from 3 separate experiments, sampled at either 92 hours (n = 23, green) or 101 hours (n = 52, red; n = 15, blue) into differentiation. Each column represents one EB. (**B**) Correlation matrix of the 40 gene expression profiles in (a); Absolute correlation values below 0.3 are blacked out. A primitive-streak gene cluster, and two later phase ectoderm/mesoderm gene clusters are marked by red squares. (**C**) Expression levels of 12 genes across the measured EBs, showing ranges between 2 and 10 C_T_ values. (**D**) Expression levels of the same genes grouped by experiment.

**Figure 3:**
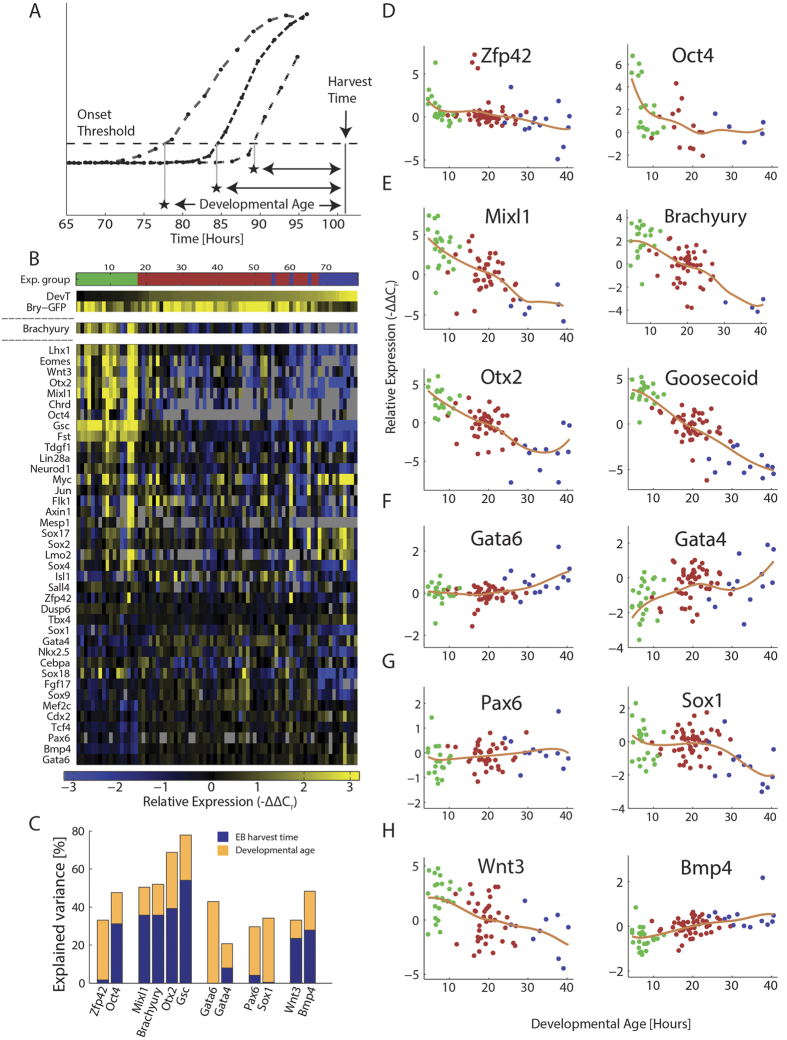
Developmental genes temporally map to a Bry-GFP defined clock. (**A**) Definition of EB Developmental Age (DevA) at the time of harvest as the time from Bry-GFP onset. **(B**) Expression of highly variable pluripotency and early development genes in individual EBs from the 3 experiments shown in Fig. 2. Columns are sorted by Bry-GFP defined Developmental Age (top row). Bry-GFP at harvest time (3^rd^ row) and Brachyury mRNA levels (4^th^ row) demonstrate the lag between mRNA and reporter levels. Most other genes show a trend corresponding with DevA. Rows are sorted by gene correlation to Brachyury mRNA expression. For each gene, the median value of the red series is subtracted for visualization. (**C**) Percent expression variance explained by Developmental Age or by EBharvest time for the 12 genes shown in Fig. 2C. (**D**–**H**) Expression of different genes plotted against developmental age. Color denotes experimental group, as in Fig. 2d. Fit curve is a local linear kernel smoothing regression on the data. Shown are pluripotency (**D**), primitive streak (**E**), mesodermal (**F**), ectodermal (**G**) and signaling (**H**) genes.

While the imaging results provide a high resolution view of spatio-temporal dynamics of early mesodermal differentiation within the EB, they do not provide information on the EB’s multi-faceted differentiation stage and the genes driving and driven by the EB progression from mesendoderm expansion to the next stage. To gain more information on these parameters within each EB, we selected 87 genes marking primitive streak, mesodermal, ectodermal and endodermal differentiation, pluripotency, and activation of signaling pathways associated with cell fate decisions. To obtain expression profiles at different EB states and developmental stages, we sampled EBs shortly after Bry-GFP onset, around peak Bry-GFP expression, and around offset. The three sampling stages, along with the observed EB temporal variability, provide a wide temporal view of gene expression profiles enabling the detection of gene expression trends. We used the Fluidigm BioMark system to profile the transcription level of each of those genes at the time of EB harvest. Gene expression was profiled for the entire EB and normalized by housekeeping genes expression, which is directly linked to the number of cells in the EB. Whole EB expression measurements can reveal coordination between signal and fate that are not cell-local. The selected gene panel includes many selective differentiation markers, allowing detection of specific developmental events related to layer formation. We have excluded from the analysis genes with low inter-EB expression variance, house-keeping genes, and genes with high sampling failure rate. Clustering the data mixed EBs with different harvest time and created functional gene clusters such as a primitive streak cluster and two later phase ectoderm/mesoderm clusters, one of which is anti-correlated to the primitive streak cluster (Fig. 2A,B). The expression of different genes varied between 2 fold and up to over 4000 fold between EBs, indicating that the sampled EBs may differ in differentiation path or stage (Fig. 2C). Some of this variability is explained by expression sampling time (Fig. 2D), yet a large portion of it, as well as batch-to-batch differences, are unexplained. To better account for this gene expression variability, we linked the imaging data and the expression profiles at the terminal time point by defining an EB-internal developmental clock, based on each EB’s Bry-GFP dynamics. In its simplest version, the clock accounts for the time passed since Bry-GFP onset, where the onset threshold was set to 300 Bry-GFP points (Fig. 3A, see Methods). Sorting the expression profiles by developmental age (DevA), we first observe that while Bry-GFP levels at harvest were sampled from both sides of peak Bry-GFP, Brachyury mRNA levels decline with DevA, suggesting the production of Brachyury decreases during this time window. This can be explained by the lag between Brachyury mRNA transcription and GFP protein production. Genes related to the primitive streak, such as Eomes, Otx2, Mixl1^27^ and Lhx1, are strongly correlated to Brachyury and either display a negative trend in their expression or switch off altogether, in synchronization with Bry-GFP onset (Fig. 3B). Later mesoderm genes such as Gata6, Mef2c and Sox9 are negatively correlated with Brachyury. Bmp4 expression rises with developmental age and is thus negatively correlated with Brachyury expression. This is consistent with in-utero dynamics, as dorsoventral axial patterning, where Bmp4 plays a dominant role, takes place after primitive streak formation^28^,^29^,^30^,^31^,^32^.

To test whether a Brachyury-GFP expression based clock is consistent with the progression rates of different germ layer molecular markers, we examined the expression of single genes as a function of the EB developmental age at the time of harvest. A tight relation between a gene’s expression and the EB’s DevA would suggest that the regulation of this gene is tightly linked to that of Brachyury. We found developmental age to be informative about the expression of several early and later genes, explaining up to 80% of their variance (Fig. 3C–H, Supplementary Figs 2 and 3). Some earlier developmental genes, including most of the primitive streak genes (Otx2, Gsc, Chrd, Bry, Mixl1, Lhx1, Eomes) and early germ-layer specific genes (Sox4, Neurod1), as well as pluripotency genes (Oct4, Zfp42), decrease expression with developmental age (Fig. 3D,E). Late mesodermal genes such as Gata4, and Gata6 show an increase over DevA (Fig. 3F), consistent with their expected expression time at late mesoderm/cardiac progenitor state^33^,^34^,^35^. Many genes do not show a clear temporal trend (Fig. 3G, Supplementary Fig. 3). These include genes expressed in multiple lineages or tissues, such as Cby1, Evx1, and Foxd3, but also some germ-layer specific genes (e.g. Pax6)^36^,^37^,^38^,^39^,^40^,^41^,^42^. Other ectodermal genes change only in the EBs pooled after Bry-GFP offset (Sox1, Fgf17). Lack of correlation with the Bry-GFP defined developmental age could indicate that either these genes are not in sync with the clock defined by mesendodermal onset, or that they do not change during our time frame.

To better interpret the inferred EB-level temporal expression profiles, we stained EBs against some of the developmental markers at different time points along the differentiation timeline (Supplementary Fig. 4). Oct4 recedes toward the periphery of the EB along time and then localizes to one region of the EB, explaining the EB-level decline in its expression. Sox1 maintains a similar level along the earlier time points, falling below detection at 114 hours. Otx2 declines monotonously in level and EB occupancy, expressed mostly at the EB outer layer, retreating to more localized regions by 114 hours. In these examples, changes in spatial occupancy as well as changes in per-cell expression both contribute to the observed EB-level expression profiles.

Few genes from the selected panel showed significant correlation with EB size preceding Bry-GFP onset (Supplementary Fig. 5). These include late mesoderm, cardiac-progenitor genes (Nkx2.5, Isl1, Mef2c and Meis1)^43^, consistent with previous reports on preferential cardiac differentiation in larger EBs^9^. Only two genes (Cebpa, Gata5) were negatively correlated with EB size. The paucity of genes whose expression was affected by size is consistent with the lack of observed correlation between size and Brachyury-GFP dynamics, suggesting that regulation of early differentiation in EBs is largely indifferent to the physical size of the embryoid body.

### Signaling perturbations of canonical Wnt and BMP pathways result in early mesendodermal onset and differentiation progression

**Figure 4:**
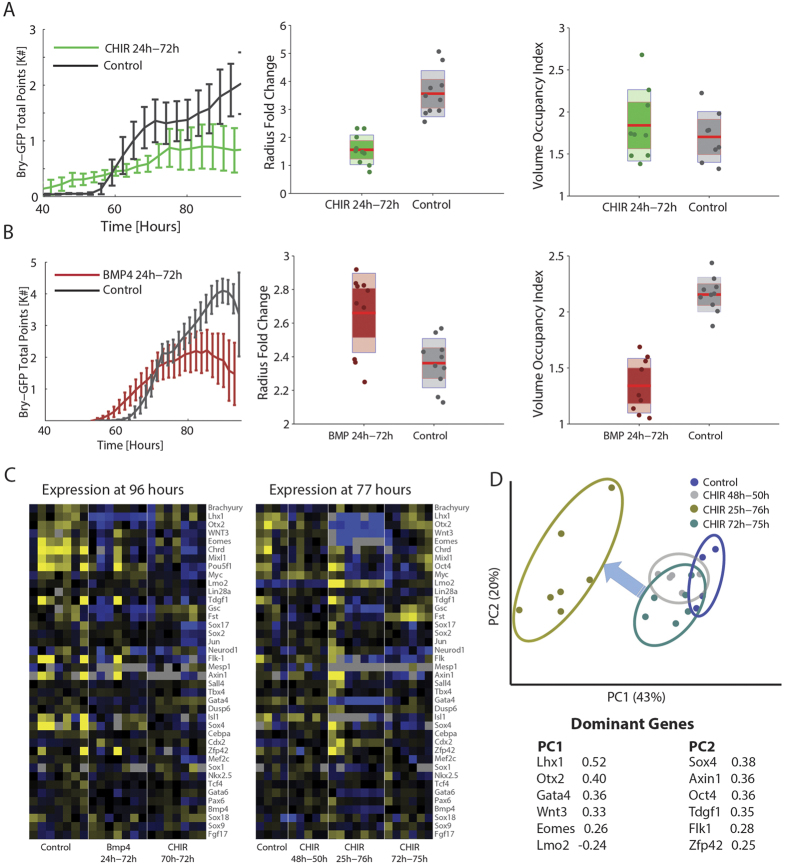
Developmental signaling perturbations affect lineages composition and timing. (**A**) Effect of Wnt activation by CHIR between 24–72 hours on Bry-GFP dynamics (green) compared to control (black), EB radius relative increase for the same experiment, and relative volume occupancy by GFP at peak Bry-GFP expression, defined as the ratio between total segmentation spots and (*EB radius*)^3^. (**B**) Effect of Bmp4 treatment between 24–72 hours shows early onset but reduced fraction of Bry+ points at peak. **(C**) Gene expression at 77 and 96 hours for Bmp4 and CHIR perturbations. Each gene is median-centered for visualization. (**D**) PCA for EBs harvested at 77 hours, comparing short and long Wnt induction periods to control. Percent explained variance is shown for each PC.

During the formation of germ layers in EBs, signaling is a major contributor to lineage specification and determination. Tampering with major signaling pathways can provide insight on the alterability of onset and development of the mesendodermal lineage, as well as that of other lineages. To test the effect of signal perturbations on Bry-GFP dynamics and on EB differentiation progression, we imaged EBs in real time under different canonical Wnt and Bmp4 signaling perturbations, and upon harvest profiled gene expression for each EB. Early activation of canonical Wnt signaling, by applying the Gsk3β inhibitor CHIR 99021 (CHIR) between 24–72 hours post differentiation, shifted Brachyury onset to an earlier time and often eliminated its spatial restriction to a single point (Fig. 4A left, Supplementary Movie 3). Bry-GFP in CHIR perturbed EBs was expressed throughout the EB at an average of 50 hours after aggregation, compared to 70 hours in the control group. Similar enhancement of Bry-GFP by Wnt3a treatment, and activation of a TCF/LEF reporter by CHIR (Supplementary Fig. 6), suggest the effect of CHIR is mainly mediated through canonical Wnt signaling^17^. Extended CHIR perturbation resulted in smaller EBs (Fig. 4A, center), explaining the low Bry-GFP peak despite the full volume occupancy (Fig. 4A, right). Primitive streak stage genes such as Otx2, Eomes, Chrd, and Lhx1 were down regulated^35^,^44^,^45^, as were some late mesodermal genes (Gata4, Gata6, Isl1)^34^ (Fig. 4C, right). Up regulated genes included later lineage genes such as Mef2c, Sox18, Lmo2 (hematopoiesis) and Neurod1 (Ectoderm), as well as Cdx2, which is expressed in various stages. Examination of the control gene expression trend indicates that CHIR perturbations were consistent with an earlier Bry-GFP onset (i.e. older developmental age) for the various CHIR down regulated genes. These findings suggest that canonical Wnt activation drove differentiation of various lineages faster in the EBs.

Bmp4 treatment between 24–72 hours shifted the onset of Bry-GFP towards an earlier time as well, but notably had less effect on EB size (Fig. 4B left, center). However, a lower occupancy of the EB by Bry-GFP at peak compared to control was observed (Fig. 4B right). In addition, primitive streak stage genes (Bry, Otx2, Lhx1, Eomes, Gsc, Cyp26a, Fgf8, Chrd), as well as pluripotency genes (Zfp42, Nanog, Oct4) were down regulated, consistent with a speedup in differentiation (Fig. 4C left, Supplementary Fig. 7). Notable upregulated genes in our panel are Fgf4, which plays a role in mesodermal patterning^46^, and Cdx2 which is expressed in all posterior germ layers at 8.5 dpc^47^. Most of the other mesodermal genes did not react to Bmp4 (Sox18, Lmo2, Mef2c, Flk1, Nkx2.5). These findings suggest that much like CHIR treatment, Bmp4 accelerates differentiation from mesendoderm to later germ layer states, but with a different derived cell composition that is reflected in different gene expression patterns.

In order to distinguish between direct and long-term effects, we also applied short (2–4 hour) CHIR perturbations at two different time periods. While short perturbations close to expression measurement (72–76 hours) are only expected to affect immediate regulatory targets, the long perturbations (24–72 hours) are expected to affect many genes through indirect interactions, as well as EB-level expression effects stemming from changes in EB cell composition. Different CHIR treatment time spans had a gradual directional effect on gene expression space, where mesodermal and primitive streak genes dominate this direction (Fig. 4D). This suggests that canonical Wnt activation had long-term effects on both direct as well as indirect targets. Short CHIR perturbation at 48–50 hours showed a much smaller effect at 77 hours, perhaps due to recovery from the perturbation (Fig. 4C,D). Neurod1 had a downward temporal trend in non-treated EBs but showed up-regulation for early CHIR and down regulation for late CHIR. Interestingly, Bmp4 was up regulated for short CHIR perturbations, consistent with its positive trend from mesendoderm onset, but slightly down regulated with long CHIR. The effects of the different CHIR perturbations suggest that canonical Wnt activation drives a gradual sequential progression in expression state rather than a sharp switch between two states.

### Signaling perturbations reveal dependency modes between signaling and target genes

**Figure 5:**
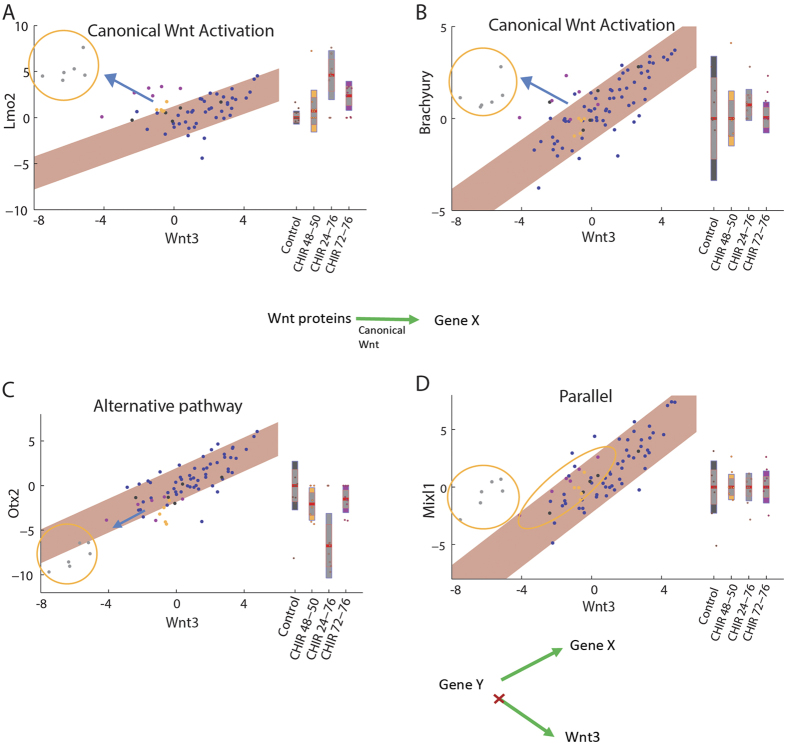
CHIR perturbation reveals different Wnt-associated modes of regulation. The expression of four target genes plotted against the expression of Wnt3 in individual EBs in −ΔΔC_T_ units. Yellow circles highlight CHIR perturbation conditions. The expression levels for the CHIR perturbations are also shown as boxplots (right). The regulation mechanism suggested for each gene from the expression pattern is shown below each plot (see text). (**A**,**B**) Lmo2 and Brachyury show positive regulation by canonical Wnt, gradually changing with activation duration. (**C**) Canonical Wnt activation keeps the same trend for Otx2, pointing to a non-canonical Wnt pathway. (**D**) Decoupling of Mixl1 from Wnt3 suggests a parallel regulation mechanism.

Different genes show positive or negative correlation with some of the signaling genes, such as Wnt3, across EBs. Such correlations could be the result of regulation by the relevant signaling pathway (e.g. canonical Wnt). Alternatively, they can reflect a common regulatory mechanism for both genes, or an independent but temporally parallel behavior. Interestingly, the activation of canonical Wnt pathway by CHIR resulted in low Wnt3 mRNA expression and, in the short term, higher Dkk1 expression in EBs, consistent with a negative feedback mechanism at the transcriptional level. This previously unreported effect better resolves as to the potential regulation pathways for different genes. As the effect of CHIR is downstream to Wnt3 in the canonical Wnt pathway, it dissociates the dependency of canonical Wnt targets from Wnt3 expression levels. However, genes affected by Wnt3 through other pathways will not be dissociated from Wnt3 level upon CHIR treatment. For example, Lmo2, highly expressed in the caudal mesodermal regions where hematopoiesis is initiated^48^, is positively correlated with Wnt3 and up regulated by CHIR (Fig. 5A). This suggests activation through the canonical Wnt pathway. Brachyury corresponds to the same dependency mode, consistent with current knowledge^49^,^50^ (Fig. 5B). For both genes, the up-regulation under CHIR contrasts with their natural trend along DevA, further supporting the activation model. Interestingly, Otx2, whose expression is confined *in-vivo* to the epiblast and later to the fore and mid-brain^51^, shows positive correlation with Wnt3, but is down regulated by CHIR, continuing the same linear trend (Fig. 5C). This could be explained by either activation by Wnt3 through a pathway other than canonical Wnt, or by expedited developmental progression of the EB, continuing the natural decline of Otx2 (Fig. 3E). Finally, Mixl1 is positively correlated with Wnt3, but CHIR treatments resulted in expression levels similar to untreated EBs. This is consistent with either a common regulation scenario or with temporally parallel independent processes (Fig. 5D). All genes correlated with Wnt3 can be categorized to one of these four modes (Supplementary Fig. 8). Notably, in many cases the response to short perturbation close to expression sampling is in the same direction as the response to the 48-hour perturbation, indicating that the identified modes reflect a gene-regulatory effect and cannot be explained solely by different EB cell composition. For example, Lmo2 shows a partial dissociation from the trend when applying short CHIR (72–76 hours) close to harvest, while a long CHIR perturbation (24–76 hours) completely dissociated the trend in the same fashion and direction as the short CHIR had (Fig. 5A). In other genes, only long CHIR treatment affected their expression, suggesting an indirect effet.

## Discussion

We have shown how Bry-GFP dynamics and selected differentiation and signaling genes in EBs follow a common pattern. Even though EBs display a natural onset time spread, we show that once Bry-GFP expression ensues, the successive dynamics follow a spread from a well-defined locus throughout the EB followed by recession back toward that locus in a predictable manner. This expands on previous observations on localized Brachyury expression from snapshot data^17^. The decoupling of Bry-GFP onset time and progression rate from the initial size of the EB suggests that mesendodermal differentiation initiates in EBs due to local events, such as ECM secretion or another self-induction mechanism^52^. It may also suggest Brachyury expansion is not regulated by size-dependent signal gradients^53^,^54^. Interestingly, later mesodermal events such as the expression of cardiac genes were dependent on size, consistent with previous reports^9^. The EB platform, in which size can be decoupled from developmental stage, allows therefore teasing out which of the processes have a dependence on physical size.

Using Bry-GFP we have defined a developmental clock for each EB. The relevance of such a clock is supported by temporal gene expression trends formed by EBs under the same conditions, regardless of their absolute Bry-GFP onset time. Multiple pluripotency and mesoderm related genes showed consistency with their ascribed developmental age, with up to 80% of their variance explained by time since Brachyury onset. This suggests that mesoderm advances through a regular differentiation program in embryoid bodies from the mesendodermal formation stage onwards. Extending the measured gene panel in the future to more ectodermal and endodermal genes would provide a fuller picture on temporal trends and synchrony between layers in this system. While whole EB expression data provides insights on developmental progression, it averages-out intra-EB spatial expression patterns. A descending expression profile, for example, could stem from either uniform decrease in all the cells, or a reduction in the fraction of cells that express the gene steadily. We have demonstrated the spatio-temporal pattern of some of the expression markers, finding good agreement with the inferred temporal expression profiles (Supplementary Fig. 4). Extending the current approach to integrate spatially-mapped single cell expression data can help resolve such ambiguities^55^,^56^,^57^.

Both Wnt and Bmp4 perturbations led to early mesendoderm marker appearance and down regulation of pluripotency and primitive streak genes, consistent with an acceleration of mesoderm progression. However, later mesodermal genes responded differently, with Wnt activation driving a more consistent cardiac lineage marker progression while Bmp4 yielded a more mixed response. The dynamic monitoring of a differentiation marker was crucial in interpreting the results, as signaling pathways assume changing roles at different stages of differentiation. For example, canonical Wnt may switch roles from maintaining pluripotency to inducing mesodermal differentiation^58^. Finally, comparing the effects of short and long Wnt activations can suggest a distinction between direct and indirect targets.

Canonical Wnt activation by CHIR unexpectedly led to down regulation of Wnt3 at the EB level, either through a previously unreported negative feedback loop, or by reducing the population of Wnt3 producing cells in the EB. This combination of pathway activation and its corresponding ligand down regulation enabled us to detect dependency modes that can be used to characterize gene relations with the Wnt3 ligand. Of particular interest is the mode where CHIR intervention maintains the gene-Wnt3 correlation, as in the case of Otx2. This mode suggests an action of Wnt3 through a pathway other than canonical Wnt, possibly through non-canonical Wnt/JNK or Wnt/calcium pathways^59^,^60^, or via the Wnt-YAP/TAZ pathway^61^,^62^. Additional experiments with combinations of Wnt3 and CHIR are needed to further validate the route of regulation. This dependency mode can be utilized to further identify genes regulated by Wnt ligands associated with the canonical pathway that are actually regulated through non-canonical Wnt pathways. This method can be generalized to other pathways by implementing two signaling perturbations, one activating the signaling pathway and the other eliminating the signaling ligand, to distinguish between effects mediated by the known pathway and other, potentially unknown alternatives.

Here we provide a novel method to temporally chart gene expression trends with respect to a developmental milestone marker in the context of a differentiation system. Using this method we were able to show how EBs follow a predictable, self-regulated mesendodermal progression, both in terms of the live marker spatio-temporal behavior and in terms of whole-EB gene expression. Our findings emphasize the potential of utilizing dynamic imaging data in the interpretation of static molecular profiling, both under wild-type progression and under signaling pathway perturbations. Adding selective fluorescence markers for earlier or later stages of differentiation and single-cell mRNA analysis can extend the spatio-temporal depiction of expression trend and improve the clock accuracy, providing a better insight into whole EB inter-related processes.

## Methods

### Cell culture

E14 Bry-GFP mouse ESCs (kindly provided by Dr. Gordon Keller^24^) were cultured using standard conditions on irradiated primary mouse embryonic fibroblasts and DMEM containing 15% fetal bovine serum, 50 ug/ml penicillin/streptomycin, 2 mM L Glutamine, 100 μM non-essential amino acids, 100 μM ß-mercaptoethanol and 10^3 ^U/mL LIF.

### EB formation and differentiation

Embryoid bodies were formed in hanging drops. Each drop contained 25ul differentiation medium (IMEM containing 20% FBS, 50 ug/ml penicillin/streptomycin, 2 mM L Glutamine, 100 μM non-essential amino acids, 100 μM ß-mercaptoethanol) with approximately 500 mES cells. Cells were aggregated over 24\48 hours before being transferred to microwells for imaging in the same medium. For signal perturbations, CHIR99021 and Bmp4 were added to the differentiation medium at 10.7 uM and 200 ng/mL final concentrations, respectively. The CHIR concentration was determined using a titration curve over Bry-GFP, 7xTCF-Strawberry EBs exposed at 24, 48 or 72 hrs into differentiation to CHIR.

### Microwells

Glass-bottom 6-well plates were treated with 50 ug/ml poly D-lysine (PDL) for 1 hour at room temperature. After rinsing with PBS, PDMS stamps were placed face down on the PDL treated glass. Agarose (0.6%) was then wicked under the stamp by pipette and the stamped plate was placed in a vacuum desiccator for 2 hrs. Following desiccation, Agarose was rewicked from the other side of the stamp and placed in the desiccator for an additional 10 minutes. Before EB transfer, microwells were sterilized by a 70% ethanol wash followed by 1 hour UV radiation.

### Microscopy

EBs were imaged using a Zeiss LSM7 inverted two-photon microscope with a 32X/0.85NA water-immersion objective. Each EB was scanned in 118 slices at 3 um intervals, totaling a depth of 354 um. Horizontal resolution was set to 112 × 112 pixels at 3.95 um per pixel. GFP was excited at 930 nm. We evaluated depth-dependent signal attenuation by comparison to Hoechst staining and Nanog:GFP signal in similar sized EBs (Supplementary Fig. 1). Imaging was carried out during the period of interest where the frame-to-frame interval was 1–2 hours. The EBs were imaged under 5% CO_2_ incubation at 37 °C.

### EB harvest

Individual EBs were harvested at a selected time point from the microwells under a Nikon stereoscopic zoom microscope. A pipet with a broad tip was used so that the entire EB mass was collected and transferred to a collection array and fixed in 4% paraformaldehyde for further processing.

### qPCR

Each EB was imaged prior to collection in 10 ul of Cells Direct Lysis Buffer (invitogen #117753–100) and stored at −80 °C. The sample lysates were then processed for mRNA expression across 96 genes. Intron-spanning PCR primers for every assay were designed using the NCBI Primer Blast tool. The designed primer sequences were ordered from IDT. The sample (single EB) preparation did not include RNA isolation, but was processed directly to a reverse transcriptase reaction (Superscript VILO cDNA synthesis kit, Applied Biosystems, Foster City, CA), which was followed by real-time PCR for targeted amplification (pre-amplification) of 96 genes by following the standard BioMark™ protocol to pre-amplify cDNA samples for 18 cycles using TaqMan^®^ PreAmp Master Mix (Applied Biosystems, Foster City, CA). qPCR reactions were performed using 96.96 BioMark™ Dynamic Arrays (Fluidigm^®^, South San Francisco, CA) enabling quantitative measurement of 96 mRNAs in each of the 96 samples per Dynamic array. This enabled 9216 (96 × 96) qPCR reactions under identical conditions^63^. DNA intercalating dye based approach (SsoFast™ EvaGreen^®^ SuperMix with Low ROX Bio-Rad Laboratories, Philadelphia, PA) was used for quantitative detection of qPCR products. Each run consisted of 30 amplification cycles (5 s at 96 °C, 20 s at 60 °C). CT values were calculated by the Real-Time PCR Analysis Software (Fluidigm).

### Image and statistical analysis

#### Visualization

The 3D data output from the microscope was visualized either by using Imaris or the FIJI 3D reconstruction feature. Each EB was qualitatively analyzed manually in order to evaluate EB viability and imaging quality.

#### Segmentation

Two photon Z-stacked images were 3D segmented using Imaris software. The nominal segment diameter was set to 8um. Segmentation objects (“points”) were filtered using a mean fluorescence intensity threshold for a diameter dependent voxel size. The parameters were chosen so that number of points will be proportional to the number of GFP expressing cells. This was verified to be correct along the differentiation timeline, with a ratio of 1.4 ± 0.3 cells per point (Supplementary Fig. 9). Segmentation results, containing 3D locations and fluorescence intensities, were further processed using MATLAB.

#### Filtering

Spatial filters were implemented to obtain clutter free data. A close-neighbor filter weeded out segmentation points with, for example, less than 5 neighbors within a 40 um Euclidean distance. A bounding box filter was used to focus on the area of interest and remove plate and agar clutter. The number of GFP points post filtering is reported throughout the text.

#### Event timing, developmental age

Each EB was fitted to a canonical form, using the following formula:


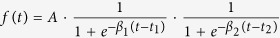


where *A* represents peak number of segmented GFP points, β_*1*_ the normalized progression rate, β_*2*_ the normalized recession rate, and *t*_*1*_, *t*_*2*_ are approximately the half peak time points for signal onset and decline, respectively. The fit was optimized for each EB using least square differences.

The model was used to smooth measurement/processing noise in the Bry-GFP profiles. An onset time for each EB was determined at the 300 Bry-GFP points threshold, proving to construct a developmental locus for Bry-GFP. This threshold was selected as the lowest that robustly filters onset time noise. Timing statistics were robust to a choice of threshold between 300–600 points. Developmental age was defined as the lag between harvest time and onset time (Fig. 3a). We considered alternative definitions for developmental age, such as the relative progression at harvest. However, this clock cannot be computed for EBs which have not reached a GFP peak by harvest time. For the same reason, defining developmental age using the canonical model’s *t*_*1*_ parameter instead of the onset time was not accurate, as the estimate of *t*_*1*_ (time of half peak value) was not reliable for those EBs.

#### Radius analysis

An image analysis algorithm was designed to find EB radius from a phase contrast image in order to facilitate processing a large number of EBs. Radius was estimated by a circumscribed circle.

#### Gene expression analysis

Fludigm mRNA data was normalized with the two most stable housekeeping genes, to compensate for differences in cell number between sampled EBs. The combination of Rock2 and Ywhaz was the most stable set of housekeeping genes, using the geNorm and NormFinder methods^64^, where both methods agreed on the same normalizing house keeping gene set. Expression was shifted gene-wise with control groups’ median gene expression, resulting in the reported −ΔΔC_T_. Hierarchical clustering was based on the Pearson correlation similarity metric. NaN values were imputed using the nearest neighbor method.

## Additional Information

**How to cite this article**: Boxman, J. *et al.* Integrated live imaging and molecular profiling of embryoid bodies reveals a synchronized progression of early differentiation. *Sci. Rep.* **6**, 31623; doi: 10.1038/srep31623 (2016).

## Supplementary Material

Supplementary Information

Supplementary Movie 1

Supplementary Movie 2

Supplementary Movie 3
