# Supplementary material for: Integrated live imaging and molecular profiling of embryoid bodies reveals a synchronized progression of early differentiation
Source: Sci Rep. 2016 Aug 17;6:31623. doi: 10.1038/srep31623 (PMC4987683; doi:10.1038/srep31623)
Supplement: Supplementary Information [file srep31623-s1.pdf]

Supplementary Information - Integrated live imaging and molecular profiling of embryoid bodies reveals a synchronized progression of early differentiation

Jonathan Boxman[1¶], Naor Sagy[1¶], Sirisha Achanta[2], Rajanikanth Vadigepalli[2*], Iftach Nachman[1*]

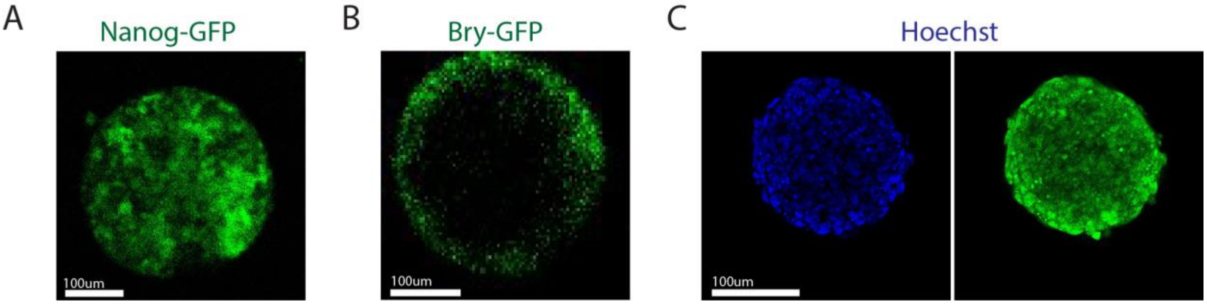

Supplementary Figure 1. Brachyury-GFP signal is limited to the EB outer shell. Comparison of signal levels at an optical slice around the EB equator in similarly sized EBs. (A) Nanog:GFP (in an undifferentiated EB composed of Nanog:GFP iPS cells). (B) Bry-GFP (C) Hoechst staining, shown for both blue and green channel detectors. Both Nanog:GFP and Hoechst show signal at the interior of the EB, while Brachyury-GFP only shows signal at the outer layer, suggesting its observed pattern is not due to signal depth-related attenuation or scattering.

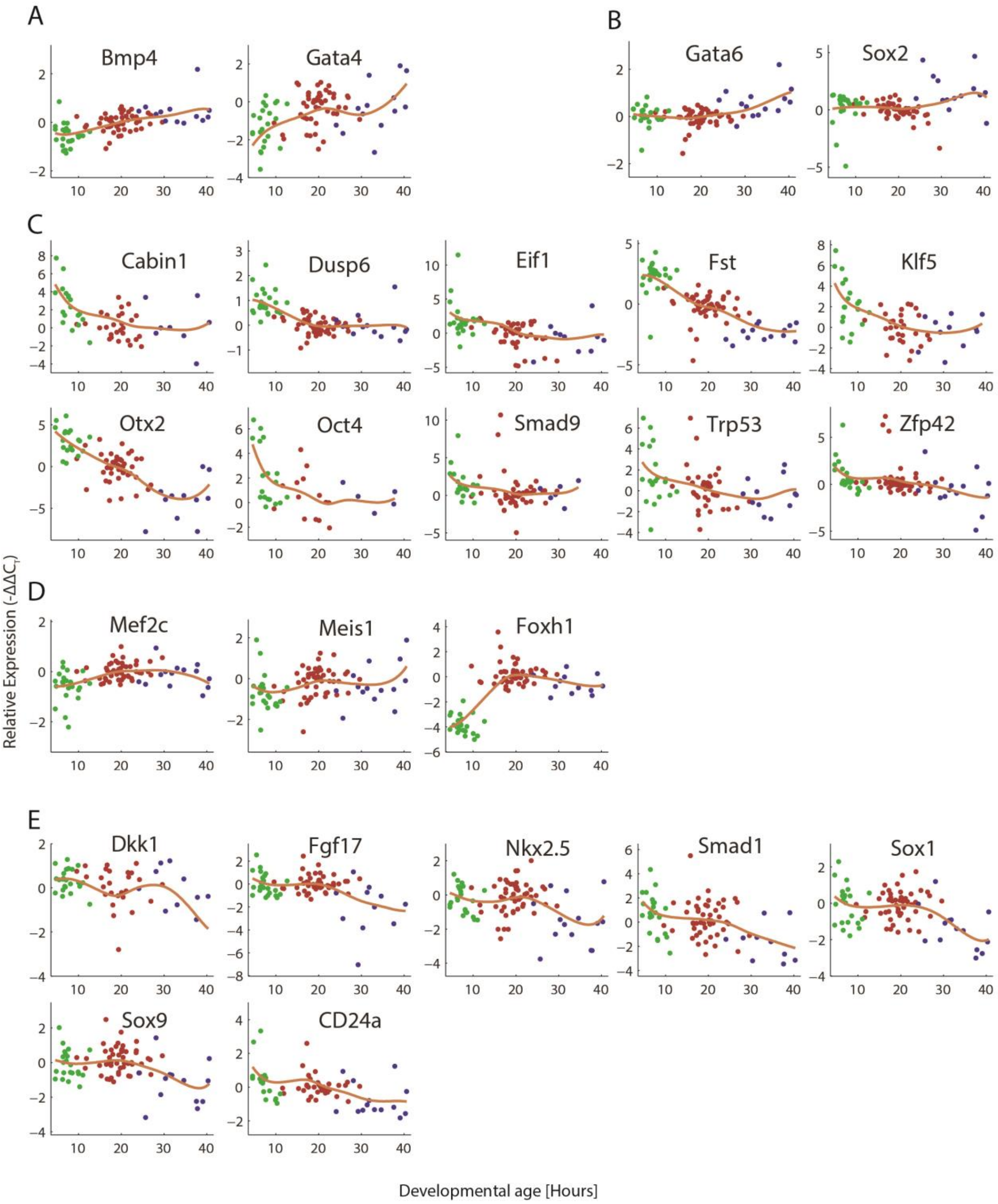

Supplementary Figure 2. EB-level gene expression plotted against developmental age, grouped by temporal trend. (a) Continuous increase, (b) Plateau and up; (c) Down and plateau; (d) Up and down;(e) Plateau and down.

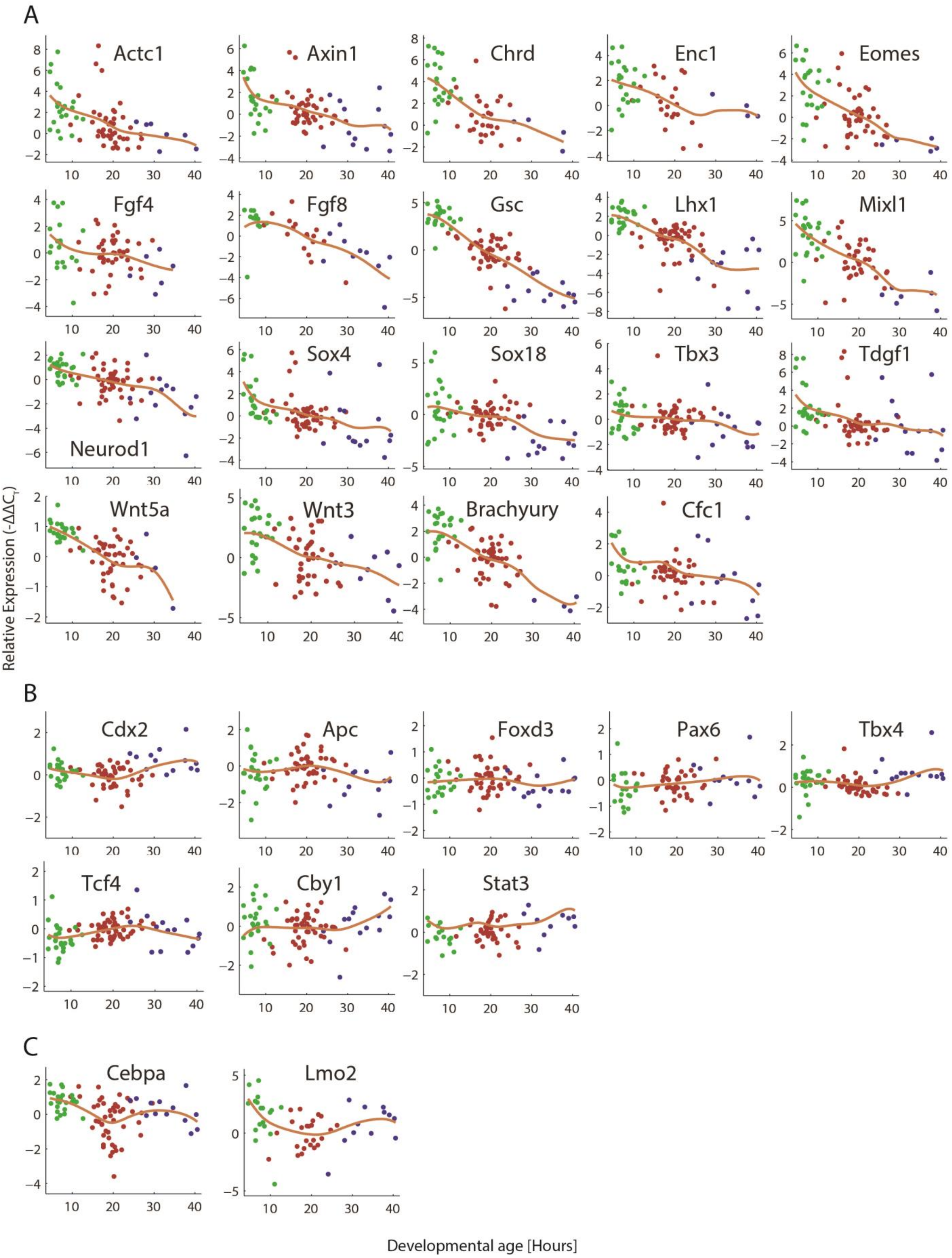

Supplementary Figure 3. EB-level gene expression plotted against developmental age, grouped by temporal trend. (a) Continuous decrease. (b) No trend. (c) Divergence.

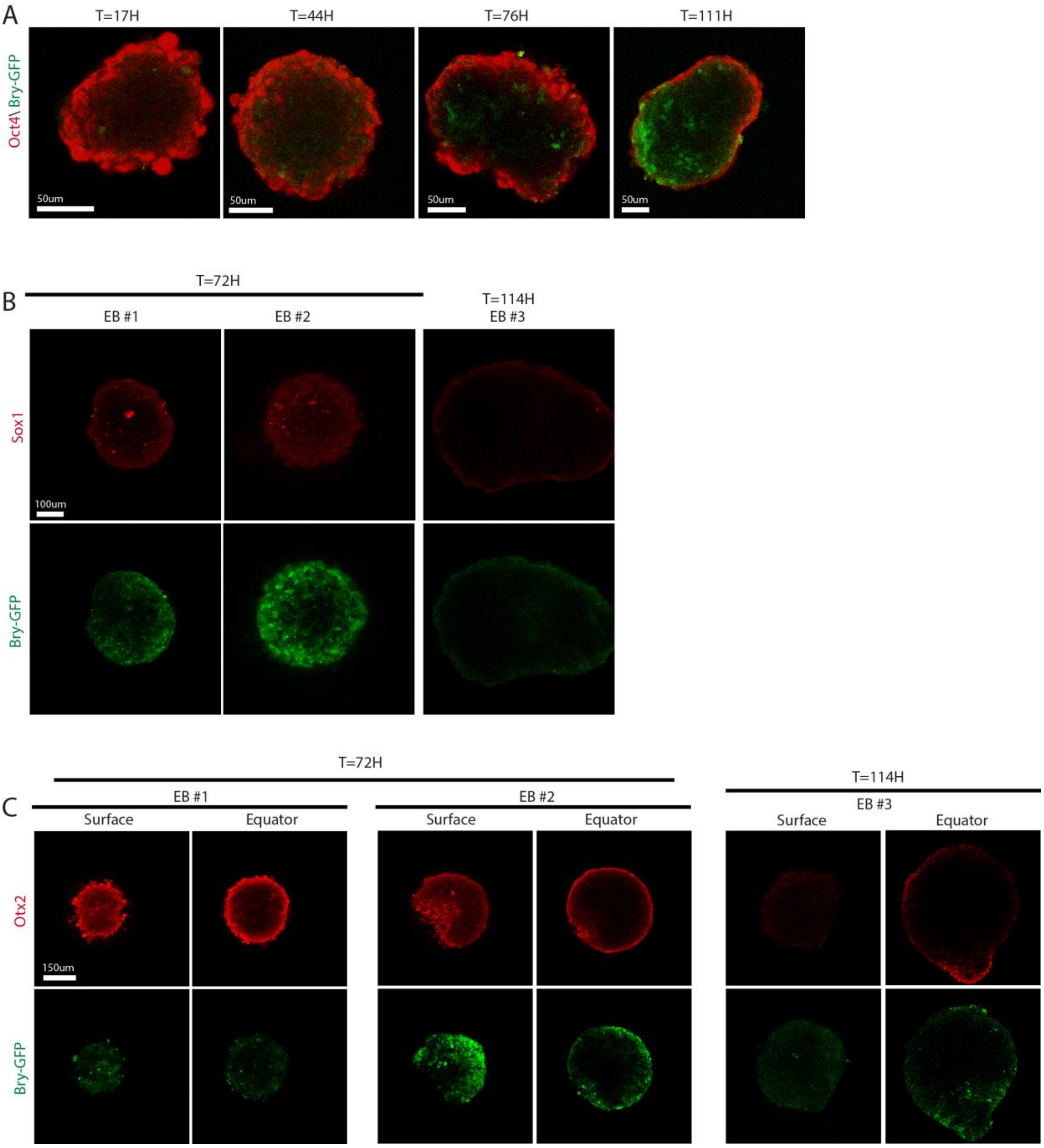

Supplementary Figure 4. EB staining of developmental markers during the differentiation timeline correspond with inferred mRNA expression profiles. Bry-GFP EBs were fixed and stained at the indicated time points along the differentiation timeline. (A) Oct4 spatial pattern recedes by 76 hours. (B) Sox1 maintains similar levels at both high- and low-DevT EBs at 76 hours, falling below detection at 114 hours. (C) Otx2 shows gradual decrease in both intensity and spatial occupancy in the EB along time. In (B,C), EB #1 and #2 at the 76hr time point are ordered by Bry-GFP, corresponding to increasing DevT at this stage (see Fig. 1D).

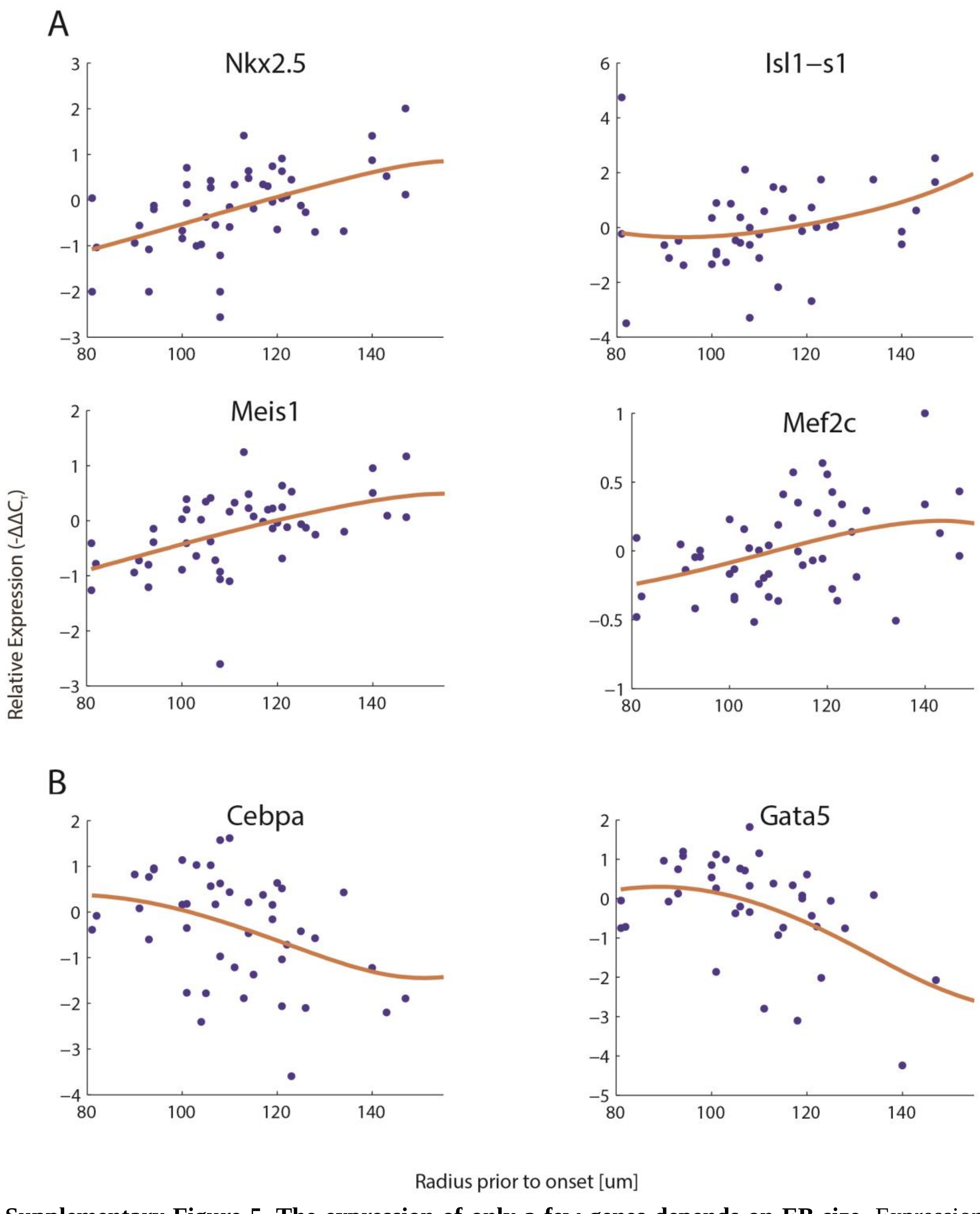

Supplementary Figure 5. The expression of only a few genes depends on EB size. Expression vs. EB radius (estimated prior to Bry-GFP onset) for genes with positive (a) or negative (b) correlation with size.

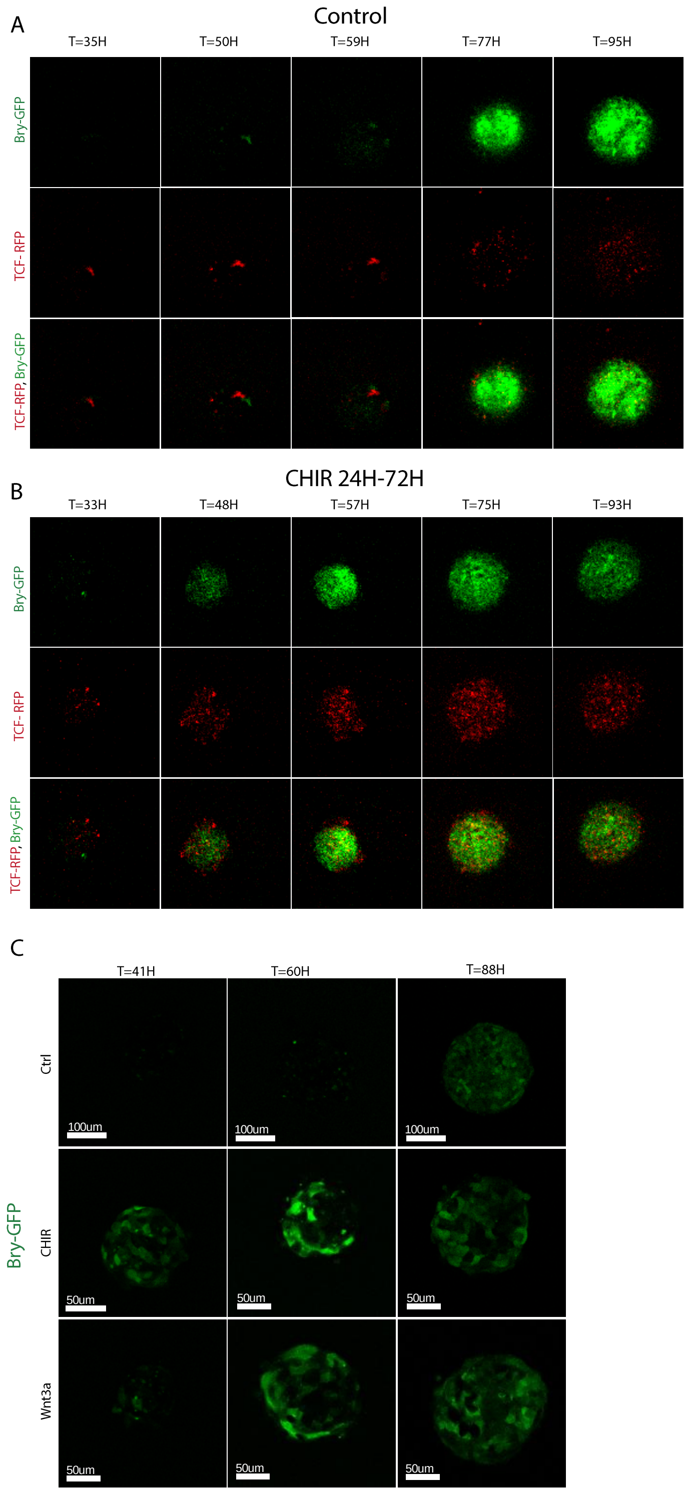

Supplementary Figure 6. CHIR treatment activates TCF/LEF reporter and modifies Bry-GFP dynamics similar to Wnt3a, indicating canonical Wnt activation. (A-B) Embryoid bodies were formed from Bry-GFP, 7xTCF-RFP(Strawberry) mES cells and differentiated under either control (no treatment) conditions (A) or CHIR treatment between 24-72 hours (B). Control (untreated) EBs show substantial Brachyury and TCF/LEF reporter activity at 77 hours post EB aggregation, while CHIR-treated EBs start showing Brachyury and TCF/LEF activity as early as 33H, and stronger TCF/LEF activity compared to control at later time points. (C) EBs were formed from Bry-GFP mES cells, and differentiated under control (top), CHIR 24-72 hrs (middle) or Wnt3a (bottom, 200 ng/mL) treatment. Both Wnt3a and CHIR treatments result in similar earlier onset of Bry-GFP.

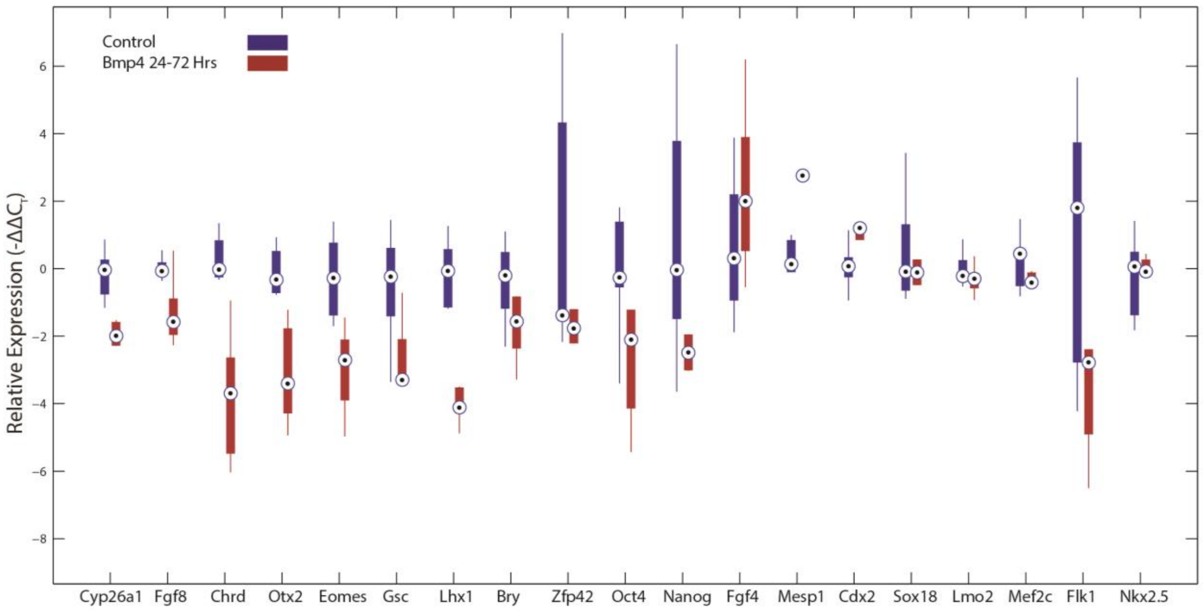

Supplementary Figure 7. Effect of Bmp4 treatment on gene expression at 96 hours. Expression of 19 genes significantly affected by Bmp4 treatment. Boxplots show the distribution of EB-level expression values in Bmp4 treated EBs (red, n=7), compared to untreated EBs (blue, n=8).

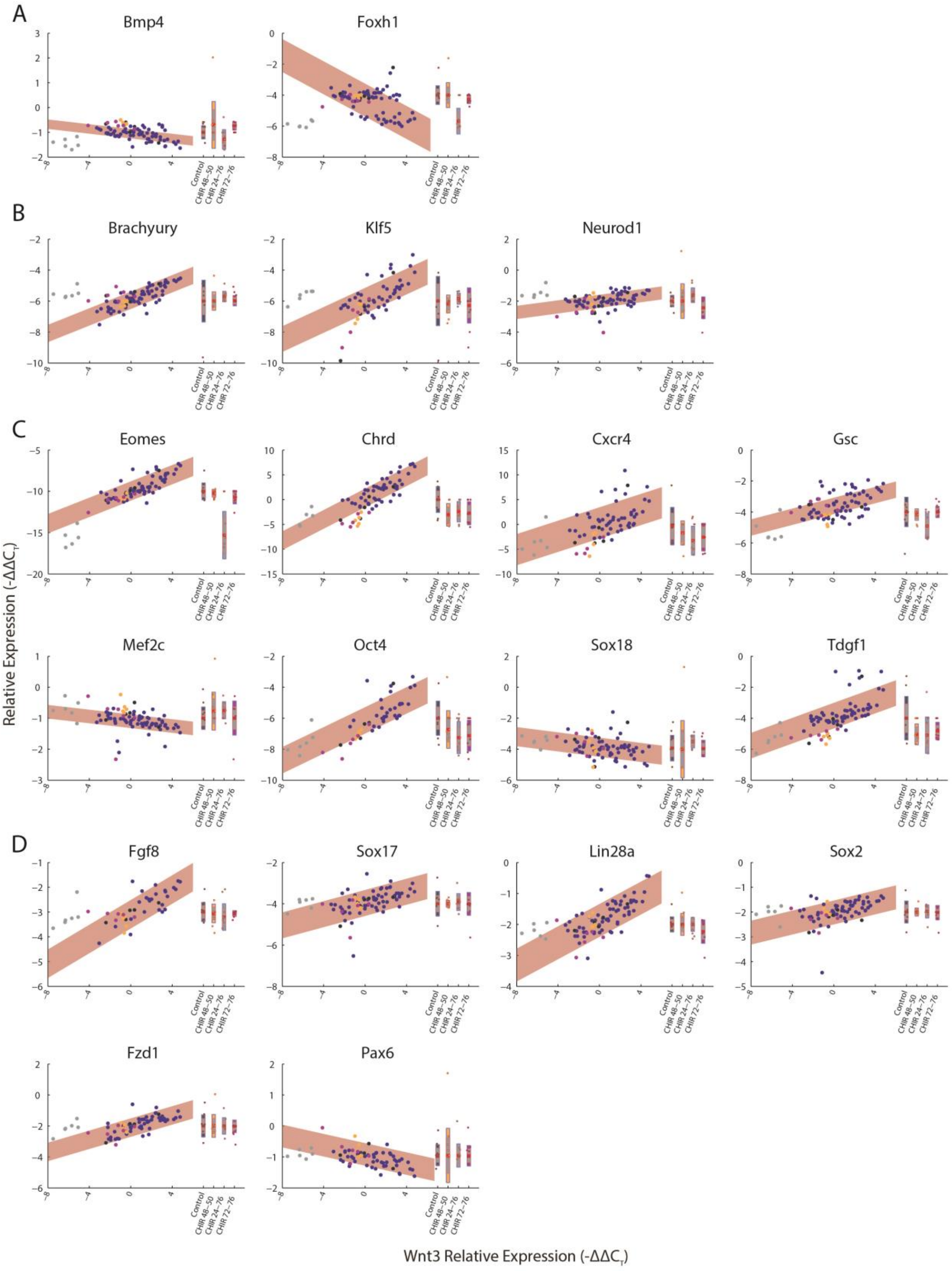

Supplementary Figure 8. Wnt3-correlated genes correspond to one of 4 regulation modes. Gene expression of different genes plotted against the expression of Wnt3 in individual EBs. The expression levels for the CHIR perturbations are also shown as boxplots (right). The plots are grouped by suggested regulation mode, as explained in Figure 5: (a) Activation mediated through canonical Wnt. (b) Inhibition mediated through canonical Wnt. (c) Activation (or, in the case of Mef2c and Sox18, inhibition) by Wnt3 through an alternative pathway. (d) Regulation in parallel to Wnt3.

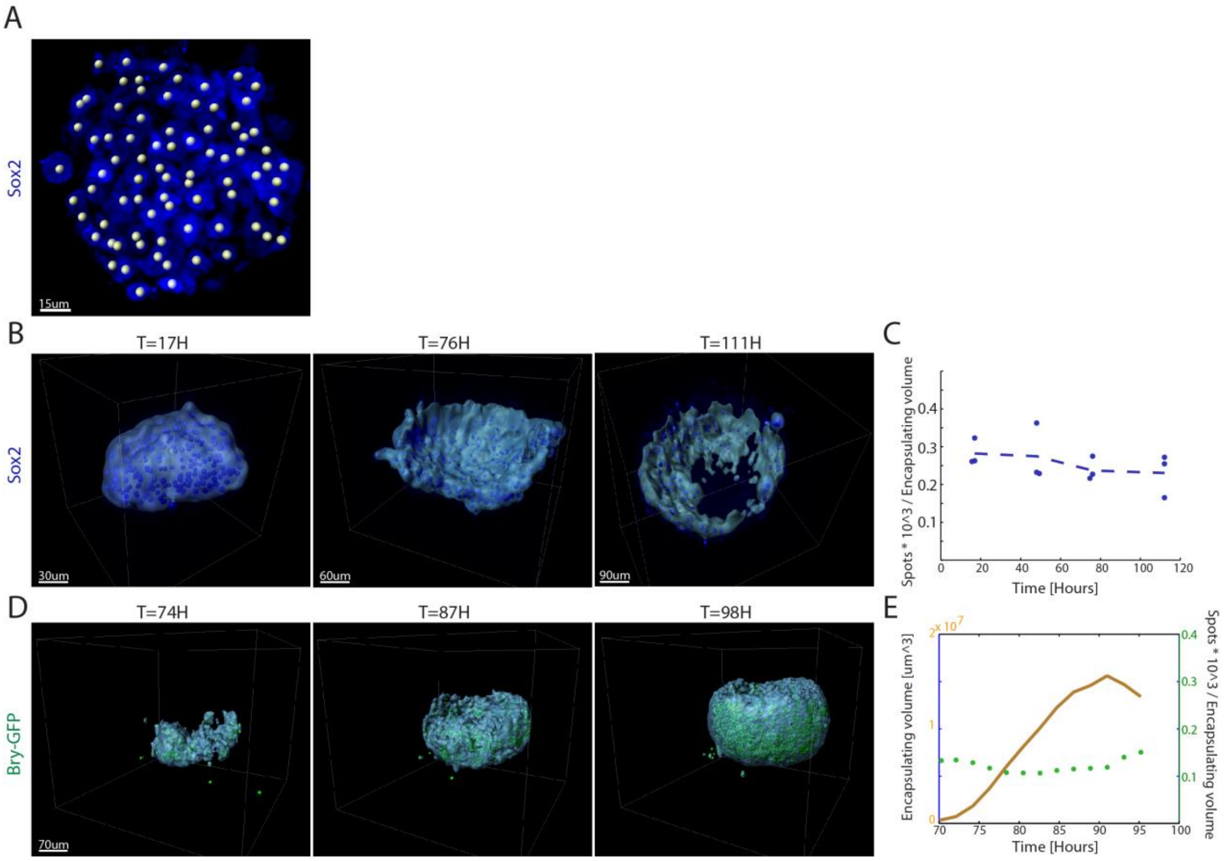

Supplementary Figure 9. Bry-GFP segmentation points are proportional to the number of Bry expressing cells. (A) Sox2 is localized to the nucleus, providing reliable cells segmentation. (B) Sox2 segmented data and encapsulating volume at 17h, 76h and 111h of differentiation. (C) The ratio between Sox2 Segmented spots and encapsulating volume over differentiation time, showing consistent volume per cell. (D) Bry-GFP segmented data and encapsulating volume at 74h, 87h and 98h. (E) The ratio between Bry segmented spots and encapsulating volume is maintained constant over differentiation time (green points), though total encapsulating volume (brown line) changes considerably. The proportionality factor between the number of cells and the number of GFP points is 1.4 ± 0.3 over our imaging time frame.

Supplementary Movie 1. Progression of Brachyury-GFP expression in an EB undergoing non-directed differentiation. Left: 3D view of GFP channel imaging data from one EB imaged between 60 – 100 hours after switch to differentiation medium. Center: Results of segmentation. Right: Total number of Bry-GFP points as a function of time, plotted on top of fit to the impulse model (see text).

Supplementary Movie 2. Comparative progression of Brachyury-GFP in 6 differentiating EBs. 3D view of GFP channel imaging data from six EBs imaged between 60 – 100 hours after switch to differentiation medium.

Supplementary Movie 3. CHIR treatment induces earlier Brachyury-GFP onset. 3D view of GFP channel imaging data from EBs imaged between 38 and 87 hours after switch to differentiation medium. Top: untreated EBs. Bottom row: EBs treated with CHIR between 24-74 hours.
